# Jejunovesical Fistula Diagnosis After Normal Vaginal Delivery: A Case Report

**DOI:** 10.31729/jnma.8407

**Published:** 2024-01-31

**Authors:** Deepak Kumar Yadav, Mahesh Bahadur Adhikari, Bipin Maharjan, Prashant Mishra, Pramesh Prasad Shrestha

**Affiliations:** 1 Department of Urology and Kidney Transplant, Nepal Mediciti Hospital, Nakhkhu Patan, Karyabinayak, Lalitpur, Nepal

**Keywords:** *case reports*, *fistula*, *jejunum*, *urinary bladder*

## Abstract

Enterovesical fistula represents an abnormal communication between the intestine and bladder. The causes are diverticulitis (56.3%), malignant tumours, which are located mainly in the intestine (20.1 %), and Crohn's disease (9.1%). Other causes include iatrogenic injury (3.2%); trauma; foreign bodies in the intestinal tract; radiotherapy; chronic appendicitis; tuberculosis; and syphilis. Normal vaginal delivery as a cause for enterovesical fistula has not been reported in many publications yet. We report a case of a 30-year-old female, who developed an jejunovesical fistula after normal vaginal delivery. It was diagnosed after diagnostic cystoscopy and computed tomography of the abdomen and pelvis. There was jejuno-vesical fistula. Resection of the segment of the jejunum with side-to- side anastomosis with bladder repair was done. A follow-up cystogram was done which showed no contrast extravasation into the peritoneum. The patient was followed up for 9 months after surgery.

## INTRODUCTION

A fistula is an abnormal or surgically made passage between a hollow or tubular organ and the body surface or between two hollow or tubular organs.^1^ Enterovesical fistula (EVF) represents an abnormal communication between the intestine and bladder. Causes of EVF have been divided into five main classes: congenital, traumatic, tumour, inflammatory, and others. The causes are diverticulitis (56.3%), malignant tumours, which are located mainly in the intestine (20.1%), and Crohn's disease (9.1%). Other causes include iatrogenic injury (3.2%), trauma, foreign bodies in the intestinal tract; radiotherapy, chronic appendicitis; tuberculosis, and syphilis.^2^

## CASE REPORT

A 30-year-old lady presented with complaints of difficulty in passing urine following 5 days after normal vaginal delivery. She also complained of abdominal distention and abdominal pain since then. She had a few episodes of vomiting which mainly contained food particles. She had increased urinary frequency ten to twelve times per day but less than 5 ml per episode associated with dysuria. There was repeated blockage of the Foley catheter and failure of trial without a catheter at a tertiary centre where she was being treated for the same issues. She is a known case of hypothyroidism under thyroxine. She had not undergone any surgery in the past.

On presentation, she was dehydrated and pale. On abdominal examination, the abdomen was distended and soft, and the gravid uterus was palpable in the lower abdomen with the tympanic note above the uterus and bowel sound was normal. There was a Foley catheter in situ, draining urine. We made a provisional diagnosis of urinary tract infection.

**Figure 1:**
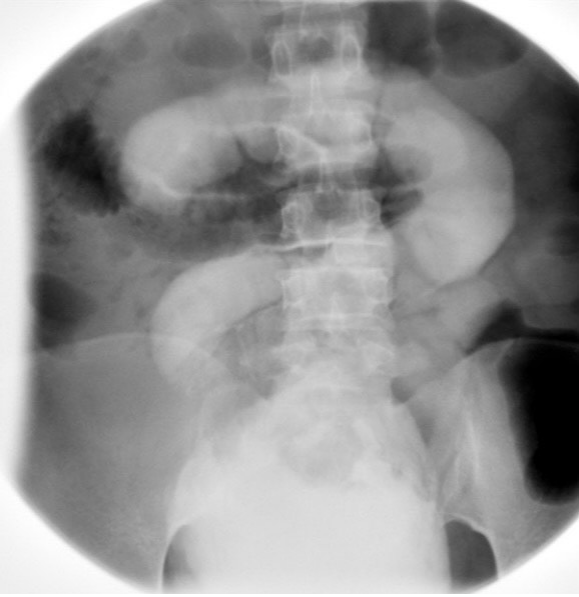
Cystogram showing abnormal communication between bowel loop in the midline likely small bowel loops and antero-superior wall of urinary bladder.

Complete blood count and renal function test were normal. A stool routine examination showed Entamoeba histolytica. Urine routine examination showed 8-10 leucocytes/hpf. Urine culture was normal. Ultrasound of the abdomen and pelvis showed a markedly thickened urinary bladder (UB) wall with trabeculations measuring 12.1 mm and a bladder filled with debris. Cystoscopy was done where the bladder wall was not visible clearly, the bladder was filled with flakes and irrigation fluid came out from the rectum. Suspecting it to be an jejunovesical fistula, a cystogram was performed and there was evidence of abnormal communication between bowel loops in the midline likely small bowel loops and the antero-superior wall of the urinary bladder (Figure 1).

**Figure 2:**
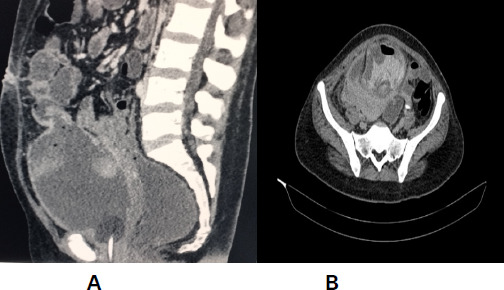
A) CECT abdomen and pelvis with enterography showing entero-vesical fistula between the dome of the urinary bladder and the adjacent ileal loop, B) CECT abdomen and pelvis with enterography showing air in the urinary bladder with the thickened posterior bladder wall.

Following this contrast enhanced computed tomography (CECT) abdomen and pelvis with enterography was performed which showed an entero-vesical fistula between the dome of the urinary bladder and adjacent ileal loop, 12.3 mm and no bowel pathology and air in the urinary bladder with the thickened posterior bladder wall (Figure 2).

**Figure 3:**
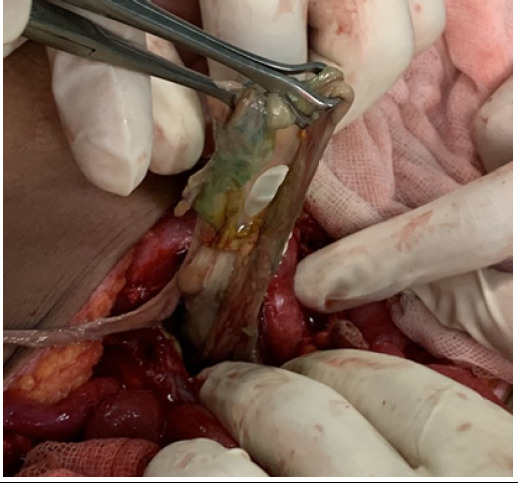
Flakes from the urinary bladder.

**Figure 4:**
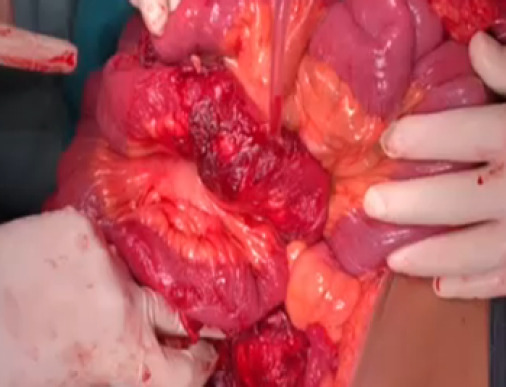
Unhealthy jejunum with fistula.

The final diagnosis of jejunovesical fistula was made and proceeded with exploratory laparotomy. Intra- operatively dense adhesion of bowel, omentum and UB with anterior abdominal wall, dense adhesion of multiple bowel loops of jejunum (approximately 30 cm) around 60 cm distal to duodenojejunal (DJ) junction with dome and posterior wall of UB with communication between UB and jejunum, UB filled with flakes, debris, purulent urine and dense adhesion of the UB with uterus was found (Figure 3) and (Figure 4).

Resection of the segment of the jejunum with side-to- side anastomosis with bladder repair was done.

**Figure 5:**
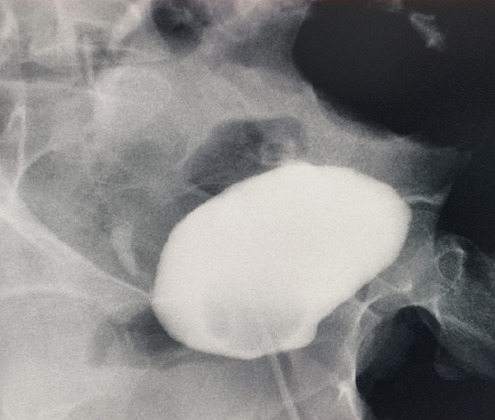
Cystogram showing no evidence of contrast extravasation into the peritoneum.

The postoperative period was uneventful. She had hypochloremic metabolic acidosis (ABG: pH-7.2, PCO_2_-10 mmHg, HCO3-3, 3 mmol/L, Cl-65mmol/L) preoperatively that was corrected. The patient was discharged on the seventh post-operative day with a Foley catheter and a follow-up cystogram was done after 2 weeks and there was no evidence of contrast extravasation into the peritoneum (Figure 5).

Foley was removed. During her follow-up for 9 months, She did not have any significant issues.

## DISCUSSION

The peak incidence of vesicoenteric fistula occurs at 55 to 65 years of age, although fistulae from Crohn's disease manifest much earlier.^3^ Symptoms of vesicoenteric fistulae may originate from the urinary or Gastrointestinal (GI) tract. However, in general, lower urinary tract symptoms are more common at presentation which include pneumaturia, frequency, urgency, suprapubic pain, recurrent urinary tract infections (UTIs), and hematuria. Pneumaturia is considered the most common presenting symptom noted in 50% to 70% of cases.^4^ GI symptoms may include fecaluria and tenesmus. Gouverneur's syndrome characterized by suprapubic pain, frequency, dysuria, and tenesmus is the main hallmark of EVF.^5^

Cystoscopy has the highest yield in identifying a potential lesion, with some type of abnormality noted on endoscopic examination in more than 90% of cases.^4^ However, the findings on cystoscopy are often nonspecific and include localized erythema, papillary, or bullous change; a definitive diagnosis using cystoscopy can be made in only 35% to 46% of cases.^6^ Cross-sectional imaging, especially computed tomography (CT) scanning, has become the imaging modality of choice. CT scanning should be performed following oral administration of contrast but before intravenous administration of contrast, to permit the detection of Gastrografin or other diluted iodinated contrast agents within the bladder. The findings on CT, which are suggestive of enterovesical fistula include (i) air in the bladder (in the absence of previous lower urinary tract instrumentation), (ii) oral contrast medium in the bladder on non-intravenous contrast- enhanced scans, (iii) presence of colonic diverticula, and (iv) bladder wall thickening adjacent to a loop of thickened intestine.^7^ Cystography and transrectal contrast studies (e.g. barium enema) are in general less likely to demonstrate the fistula.^8^ Similarly in this case, cystoscopy lead to the initial diagnosis of enterovesical fistula and jejunovesical fistula was confirmed by contrast enhanced computed tomography (CECT) Abdomen And Pelvis With Enterography. Cystography and transrectal contrast studies (e.g., barium enema) are in general less likely to demonstrate the fistula.^8^

In nontoxic, minimally symptomatic patients with nonmalignant causes of enterovesical fistulae, a trial of medical therapy including intravenous total parenteral nutrition, bowel rest, and antibiotics may be warranted. This may be the preferred initial approach, especially in patients with Crohn's disease, in whom the notion of immediate exploratory laparotomy and bowel resection is often discouraged because of the chronic relapsing nature of the disease.^9^ The goal of operative management is to separate and close the involved organs with minimal anatomic disruption and normal long-term function of both systems. The choice of whether to proceed with a one-stage or two-stage repair is influenced by the location and cause of the fistula, the patient's general condition, the presence of a pelvic abscess, and the presence of colonic obstruction. Patients with an inflammatory cause of the fistula but without gross contamination can be treated with a one-stage procedure, whereas those with an unprepared bowel, gross contamination, or abscess may require a multistage procedure.^10^

Several studies have focused on enterovesical fistula related to bowel disorders. However, in this instance, bowel pathologies were excluded. As far as we are aware, there is no documented instance of a jejunovesical fistula diagnosed post normal vaginal delivery. Furthermore, the specific cause of the jejunovesical fistula in this case remains undetermined, and we cannot definitively attribute it to complications arising from vaginal delivery. This case report also prompts consideration regarding the potential causation of vaginal delivery for the development of jejunovesical fistula.
